# 3′,6′-Bis(diethyl­amino)-2-[(*E*)-2-(4-hy­droxy-3-meth­oxy­benzyl­idene­amino)­eth­yl]spiro­[isoindoline-1,9′-xanthen]-3-one ethanol monosolvate

**DOI:** 10.1107/S1600536812016741

**Published:** 2012-04-21

**Authors:** Zhen Wei, Jinlong Guo, Xujun Zheng, Shunwei Chen, Qun Wan

**Affiliations:** a Department of Basic Science, Tianjin Agriculturial University, Tianjin Jinjing Road No. 22, Tianjin 300384, People’s Republic of China

## Abstract

In the title compound, C_38_H_42_N_4_O_4_·C_2_H_6_O, prepared *via* a spiro­lactam ring-formation reaction in a rhodamine dye, the xanthene ring system is approximately planar (r.m.s. deviation = 0.0014Å) and subtends dihedral angles of 88.10 (3) and 86.92 (4)° with the spiro­lactam (r.m.s. deviations = 0.0012 Å) and benzene rings, respectively. The crystal structure consists of chains parallel to [-101], formed *via* O—H⋯O inter­actions.

## Related literature

For related structures and background to rhodamine-based dyes, see: Xu *et al.*(2010*a* ▶,*b* ▶); Zhang *et al.* (2008 ▶); Tian & Peng (2008 ▶); Kwon *et al.* (2005 ▶); Wu *et al.*(2007 ▶). 
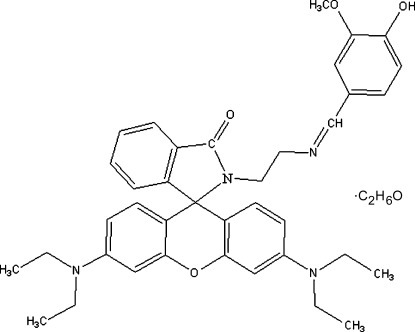


## Experimental

### 

#### Crystal data

C_38_H_42_N_4_O_4_·C_2_H_6_O
*M* *_r_* = 664.82
Monoclinic, 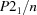

*a* = 16.674 (4) Å
*b* = 12.197 (3) Å
*c* = 17.936 (4) Å
β = 96.445 (4)°
*V* = 3624.5 (15) Å^3^
*Z* = 4
Mo *K*α radiation
μ = 0.08 mm^−1^
*T* = 113 K
0.20 × 0.12 × 0.10 mm

#### Data collection

Rigaku Saturn724+ diffractometer
Absorption correction: multi-scan (*CrystalClear*; Rigaku, 2008 ▶) *T*_min_ = 0.984, *T*_max_ = 0.992
45486 measured reflections
8576 independent reflections
7385 reflections with *I* > 2σ(*I*)
*R*_int_ = 0.043

#### Refinement

*R*[*F* ^2^ > 2σ(*F* ^2^)] = 0.049
*wR*(*F* ^2^) = 0.119
*S* = 1.10
8576 reflections
450 parameters
H-atom parameters constrained
Δρ_max_ = 0.32 e Å^−3^
Δρ_min_ = −0.21 e Å^−3^

### 

Data collection: *CrystalClear* (Rigaku, 2008 ▶); cell refinement: *CrystalClear*; data reduction: *CrystalClear*; program(s) used to solve structure: *SHELXS97* (Sheldrick, 2008 ▶); program(s) used to refine structure: *SHELXL97* (Sheldrick, 2008 ▶); molecular graphics: *SHELXTL* (Sheldrick, 2008 ▶); software used to prepare material for publication: *CrystalStructure* (Rigaku, 2008 ▶).

## Supplementary Material

Crystal structure: contains datablock(s) I, global. DOI: 10.1107/S1600536812016741/bg2444sup1.cif

Structure factors: contains datablock(s) I. DOI: 10.1107/S1600536812016741/bg2444Isup2.hkl

Supplementary material file. DOI: 10.1107/S1600536812016741/bg2444Isup3.cml

Additional supplementary materials:  crystallographic information; 3D view; checkCIF report

## Figures and Tables

**Table 1:** Hydrogen-bond geometry (Å, °)

*D*—H⋯*A*	*D*—H	H⋯*A*	*D*⋯*A*	*D*—H⋯*A*
O3—H3⋯O5^i^	0.84	1.96	2.7052 (15)	148
O5—H5*A*⋯O2	0.84	1.93	2.7552 (14)	166

Symmetry code: (i) 
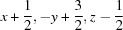
.

**Fig. 1.**
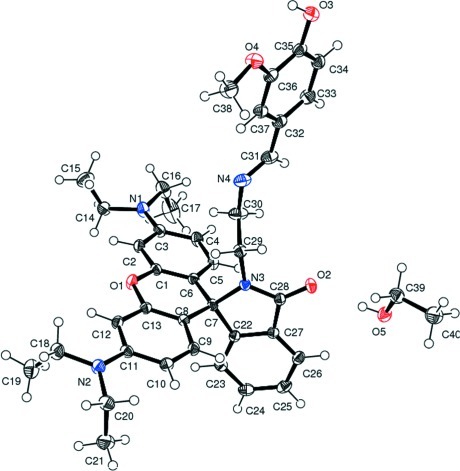
The molecular structure of the title compound with the atom numbering scheme. Displacement ellipsoids are drawn at the 30% probability level.

**Fig. 2.**
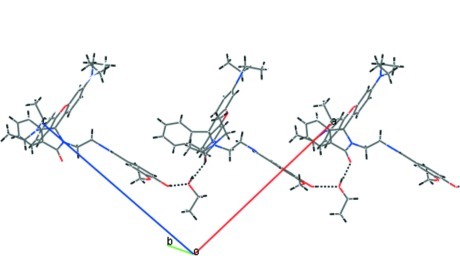
The one dimentional structure formed by the inter-molecular hydrogen bonds drawn in dashed lines

## supplementary crystallographic information

### Comment

Rhodamine-based dyes have been widely used for conjugation with biomolecules, owing to their excellent spectroscopic properties such as large molar extinction coefficient and high fluorescence quantum yields. Moreover, it is well known that many derivatives of Rhodamine undergo equilibrium between spirolactam and an open ring amide, and both conformations behave with different spectroscopic properties, for what Rhodamine-based dyes have been widely used as sensing materials (Kwon *et al.*, 2005). Detailed information on their molecular and crystal structures is necessary to understand their photophysical and photochemical properties. In the title compound [(C_38_H_42_N_4_O_4_)], the xanthene and the spirolactam rings are almost planar (r.m.s. deviations from the mean plane, 0.0014Å and 0.0012 Å, respectively), with the former ring forming dihedral angles of 88.10 (3)° to the spirolactam ring and 86.92 (4)°. to the benzene ring. The crystal structure consists of one-dimensional chains parallel to [101], formed *via* O3—H3···O5, O5—H5A···O2 interactions (Table 1).

### Experimental

*N*-(rhodamine-6 G)lactam-ethylenediamine (5*m* mol) was dissolved in 50 ml of ethanol, followed by addition of 3-methoxy-4-hydroxybenzaldehyde(5*m* mol). The solution was stirred and refluxed for 10 h. The white precipitate was filtrated and disolved in ethanol. Single crystals suitable for X-ray measurements were obtained at room temperature by slow evaporation of this solution.

### Refinement

All H atoms were seen in the final diffreence map, but further replaced at their expected positions and treated as riding on their parent atoms, with C—H = 0.93 Å for the aromatic, 0.96 Å for the methyl and C—H= 0.97 Å for methylene H atoms, and O—H: 0.84Å. In al cases *U*_iso_(H)= x×*U*_eq_(Host) with x = 1.2 except for methyl groups in which x = 1.5.

### Crystal data

**Table:**

C_38_H_42_N_4_O_4_·C_2_H_6_O	*F*(000) = 1424
*M**_r_* = 664.82	*D*_x_ = 1.218 Mg m^−^^3^
Monoclinic, *P*2_1_/*n*	Mo *K*α radiation, λ = 0.71073 Å
Hall symbol: -P 2yn	Cell parameters from 10662 reflections
*a* = 16.674 (4) Å	θ = 1.7–27.9°
*b* = 12.197 (3) Å	µ = 0.08 mm^−^^1^
*c* = 17.936 (4) Å	*T* = 113 K
β = 96.445 (4)°	Prism, colourless
*V* = 3624.5 (15) Å^3^	0.20 × 0.12 × 0.10 mm
*Z* = 4	

### Data collection

**Table:**

Rigaku Saturn724+ diffractometer	8576 independent reflections
Radiation source: fine-focus sealed tube	7385 reflections with *I* > 2σ(*I*)
Graphite monochromator	*R*_int_ = 0.043
Detector resolution: 28.5714 pixels mm^-1^	θ_max_ = 27.8°, θ_min_ = 1.6°
profile data from ω–scans	*h* = −21→20
Absorption correction: multi-scan (*CrystalClear*; Rigaku, 2008)	*k* = −16→15
*T*_min_ = 0.984, *T*_max_ = 0.992	*l* = −23→22
45486 measured reflections	

### Refinement

**Table:**

Refinement on *F*^2^	Primary atom site location: structure-invariant direct methods
Least-squares matrix: full	Secondary atom site location: difference Fourier map
*R*[*F*^2^ > 2σ(*F*^2^)] = 0.049	Hydrogen site location: inferred from neighbouring sites
*wR*(*F*^2^) = 0.119	H-atom parameters constrained
*S* = 1.10	*w* = 1/[σ^2^(*F*_o_^2^) + (0.0532*P*)^2^ + 0.5173*P*] where *P* = (*F*_o_^2^ + 2*F*_c_^2^)/3
8576 reflections	(Δ/σ)_max_ < 0.001
450 parameters	Δρ_max_ = 0.32 e Å^−^^3^
0 restraints	Δρ_min_ = −0.21 e Å^−^^3^

### Special details

**Table:**

Geometry. All esds (except the esd in the dihedral angle between two l.s. planes) are estimated using the full covariance matrix. The cell esds are taken into account individually in the estimation of esds in distances, angles and torsion angles; correlations between esds in cell parameters are only used when they are defined by crystal symmetry. An approximate (isotropic) treatment of cell esds is used for estimating esds involving l.s. planes.
Refinement. Refinement of F^2^ against ALL reflections. The weighted R-factor wR and goodness of fit S are based on F^2^, conventional R-factors R are based on F, with F set to zero for negative F^2^. The threshold expression of F^2^ > 2sigma(F^2^) is used only for calculating R-factors(gt) etc. and is not relevant to the choice of reflections for refinement. R-factors based on F^2^ are statistically about twice as large as those based on F, and R- factors based on ALL data will be even larger.

### Fractional atomic coordinates and isotropic or equivalent isotropic displacement parameters (Å^2^)

**Table:**

	*x*	*y*	*z*	*U*_iso_*/*U*_eq_	
O1	0.70816 (5)	0.41422 (7)	0.34461 (6)	0.0243 (2)	
O2	0.45673 (6)	0.76065 (8)	0.19829 (5)	0.0257 (2)	
O3	0.72460 (6)	0.76249 (8)	−0.25561 (5)	0.0281 (2)	
H3	0.7427	0.7031	−0.2705	0.042*	
O4	0.70162 (6)	0.56284 (8)	−0.19741 (5)	0.0284 (2)	
N1	0.91108 (7)	0.68357 (10)	0.39924 (7)	0.0279 (3)	
N2	0.53054 (7)	0.10821 (9)	0.31672 (7)	0.0238 (3)	
N3	0.54525 (6)	0.62774 (9)	0.24766 (6)	0.0171 (2)	
N4	0.66575 (7)	0.64858 (11)	0.08517 (7)	0.0296 (3)	
C1	0.72358 (8)	0.52500 (10)	0.35283 (7)	0.0185 (3)	
C2	0.80483 (8)	0.55002 (11)	0.37132 (7)	0.0210 (3)	
H2	0.8432	0.4923	0.3787	0.025*	
C3	0.83095 (8)	0.65951 (11)	0.37922 (7)	0.0217 (3)	
C4	0.77103 (8)	0.74200 (11)	0.36664 (8)	0.0239 (3)	
H4A	0.7862	0.8171	0.3700	0.029*	
C5	0.69080 (8)	0.71404 (11)	0.34955 (7)	0.0215 (3)	
H5	0.6519	0.7711	0.3423	0.026*	
C6	0.66416 (7)	0.60495 (10)	0.34241 (7)	0.0171 (3)	
C7	0.57572 (7)	0.57688 (10)	0.32152 (7)	0.0168 (3)	
C8	0.56436 (7)	0.45356 (10)	0.31992 (7)	0.0172 (3)	
C9	0.48720 (8)	0.40554 (11)	0.30829 (7)	0.0197 (3)	
H9	0.4414	0.4522	0.3007	0.024*	
C10	0.47538 (8)	0.29366 (11)	0.30742 (7)	0.0209 (3)	
H10	0.4220	0.2653	0.2997	0.025*	
C11	0.54168 (8)	0.22038 (10)	0.31793 (7)	0.0193 (3)	
C12	0.61892 (8)	0.26706 (10)	0.32955 (7)	0.0200 (3)	
H12	0.6650	0.2209	0.3368	0.024*	
C13	0.62862 (7)	0.38069 (10)	0.33055 (7)	0.0181 (3)	
C14	0.96754 (8)	0.59920 (12)	0.42960 (8)	0.0268 (3)	
H14A	0.9384	0.5463	0.4586	0.032*	
H14B	1.0101	0.6340	0.4648	0.032*	
C15	1.00743 (9)	0.53699 (15)	0.37037 (9)	0.0380 (4)	
H15A	0.9662	0.4977	0.3374	0.057*	
H15B	1.0462	0.4843	0.3948	0.057*	
H15C	1.0356	0.5888	0.3406	0.057*	
C16	0.94234 (9)	0.79470 (13)	0.39605 (9)	0.0361 (4)	
H16A	0.9114	0.8341	0.3540	0.043*	
H16B	0.9993	0.7913	0.3855	0.043*	
C17	0.93788 (13)	0.85921 (15)	0.46778 (11)	0.0575 (6)	
H17A	0.8812	0.8693	0.4760	0.086*	
H17B	0.9634	0.9310	0.4635	0.086*	
H17C	0.9661	0.8190	0.5102	0.086*	
C18	0.59972 (9)	0.03336 (11)	0.32402 (8)	0.0277 (3)	
H18A	0.6422	0.0644	0.2959	0.033*	
H18B	0.5826	−0.0373	0.3002	0.033*	
C19	0.63582 (10)	0.01127 (14)	0.40393 (9)	0.0370 (4)	
H19A	0.6489	0.0810	0.4296	0.055*	
H19B	0.6851	−0.0324	0.4035	0.055*	
H19C	0.5968	−0.0291	0.4304	0.055*	
C20	0.45004 (9)	0.06076 (11)	0.31775 (8)	0.0270 (3)	
H20A	0.4513	−0.0164	0.3007	0.032*	
H20B	0.4117	0.1010	0.2815	0.032*	
C21	0.41881 (10)	0.06364 (13)	0.39453 (9)	0.0366 (4)	
H21A	0.4540	0.0192	0.4300	0.055*	
H21B	0.3638	0.0340	0.3904	0.055*	
H21C	0.4185	0.1395	0.4125	0.055*	
C22	0.52088 (7)	0.63452 (10)	0.37207 (7)	0.0169 (3)	
C23	0.51696 (8)	0.62006 (11)	0.44828 (7)	0.0216 (3)	
H23	0.5518	0.5701	0.4767	0.026*	
C24	0.46017 (9)	0.68132 (12)	0.48185 (8)	0.0261 (3)	
H24	0.4563	0.6729	0.5340	0.031*	
C25	0.40883 (8)	0.75484 (12)	0.44026 (8)	0.0263 (3)	
H25	0.3702	0.7950	0.4643	0.032*	
C26	0.41351 (8)	0.76991 (11)	0.36424 (8)	0.0228 (3)	
H26	0.3791	0.8204	0.3358	0.027*	
C27	0.47024 (7)	0.70856 (10)	0.33102 (7)	0.0181 (3)	
C28	0.48748 (7)	0.70542 (10)	0.25194 (7)	0.0188 (3)	
C29	0.57599 (8)	0.59905 (11)	0.17751 (7)	0.0216 (3)	
H29A	0.5332	0.6123	0.1356	0.026*	
H29B	0.5889	0.5198	0.1781	0.026*	
C30	0.65106 (9)	0.66352 (14)	0.16326 (8)	0.0326 (4)	
H30A	0.6431	0.7423	0.1735	0.039*	
H30B	0.6982	0.6372	0.1971	0.039*	
C31	0.67072 (8)	0.73611 (13)	0.04736 (8)	0.0269 (3)	
H31	0.6655	0.8039	0.0724	0.032*	
C32	0.68390 (8)	0.73984 (12)	−0.03207 (8)	0.0237 (3)	
C33	0.69533 (9)	0.84087 (12)	−0.06541 (8)	0.0266 (3)	
H33	0.6938	0.9060	−0.0366	0.032*	
C34	0.70888 (8)	0.84776 (12)	−0.14018 (8)	0.0255 (3)	
H34	0.7161	0.9172	−0.1624	0.031*	
C35	0.71177 (8)	0.75289 (12)	−0.18223 (8)	0.0228 (3)	
C36	0.69883 (8)	0.65032 (11)	−0.14962 (8)	0.0222 (3)	
C37	0.68563 (8)	0.64348 (12)	−0.07481 (8)	0.0234 (3)	
H37	0.6778	0.5741	−0.0526	0.028*	
C38	0.67573 (10)	0.45925 (12)	−0.17106 (9)	0.0336 (4)	
H38A	0.7124	0.4361	−0.1274	0.050*	
H38B	0.6761	0.4045	−0.2110	0.050*	
H38C	0.6209	0.4663	−0.1568	0.050*	
O5	0.31286 (6)	0.87055 (9)	0.16462 (5)	0.0278 (2)	
H5A	0.3581	0.8447	0.1812	0.042*	
C39	0.29463 (9)	0.84136 (13)	0.08727 (8)	0.0290 (3)	
H39A	0.3038	0.7619	0.0808	0.035*	
H39B	0.3306	0.8819	0.0566	0.035*	
C40	0.20807 (9)	0.86893 (15)	0.06150 (9)	0.0371 (4)	
H40A	0.1727	0.8273	0.0912	0.056*	
H40B	0.1959	0.8499	0.0084	0.056*	
H40C	0.1993	0.9476	0.0682	0.056*	

### Atomic displacement parameters (Å^2^)

**Table:**

	*U*^11^	*U*^22^	*U*^33^	*U*^12^	*U*^13^	*U*^23^
O1	0.0147 (5)	0.0144 (5)	0.0434 (6)	−0.0003 (4)	0.0010 (4)	0.0004 (4)
O2	0.0234 (5)	0.0265 (5)	0.0263 (5)	0.0051 (4)	−0.0012 (4)	0.0095 (4)
O3	0.0307 (6)	0.0294 (6)	0.0247 (5)	0.0006 (4)	0.0055 (4)	0.0024 (4)
O4	0.0348 (6)	0.0240 (5)	0.0277 (5)	−0.0042 (4)	0.0088 (4)	−0.0042 (4)
N1	0.0187 (6)	0.0229 (6)	0.0401 (7)	−0.0045 (5)	−0.0060 (5)	0.0068 (5)
N2	0.0228 (6)	0.0165 (6)	0.0320 (7)	−0.0016 (5)	0.0029 (5)	−0.0009 (5)
N3	0.0175 (5)	0.0176 (5)	0.0158 (5)	0.0012 (4)	0.0009 (4)	0.0010 (4)
N4	0.0242 (6)	0.0417 (8)	0.0238 (6)	−0.0021 (5)	0.0067 (5)	−0.0018 (5)
C1	0.0188 (6)	0.0152 (6)	0.0214 (7)	−0.0003 (5)	0.0021 (5)	0.0024 (5)
C2	0.0175 (6)	0.0188 (6)	0.0263 (7)	0.0017 (5)	0.0005 (5)	0.0029 (5)
C3	0.0192 (7)	0.0223 (7)	0.0226 (7)	−0.0023 (5)	−0.0018 (5)	0.0033 (5)
C4	0.0241 (7)	0.0163 (6)	0.0297 (8)	−0.0033 (5)	−0.0039 (6)	0.0017 (5)
C5	0.0212 (7)	0.0174 (6)	0.0249 (7)	0.0023 (5)	−0.0019 (5)	0.0010 (5)
C6	0.0165 (6)	0.0177 (6)	0.0168 (6)	0.0002 (5)	0.0008 (5)	0.0010 (5)
C7	0.0162 (6)	0.0176 (6)	0.0162 (6)	0.0015 (5)	−0.0005 (5)	0.0023 (5)
C8	0.0182 (6)	0.0173 (6)	0.0160 (6)	0.0001 (5)	0.0016 (5)	0.0009 (5)
C9	0.0171 (6)	0.0202 (6)	0.0213 (7)	0.0019 (5)	−0.0004 (5)	0.0017 (5)
C10	0.0174 (6)	0.0211 (7)	0.0235 (7)	−0.0029 (5)	−0.0003 (5)	0.0000 (5)
C11	0.0236 (7)	0.0160 (6)	0.0186 (7)	−0.0016 (5)	0.0033 (5)	−0.0001 (5)
C12	0.0173 (6)	0.0167 (6)	0.0264 (7)	0.0025 (5)	0.0037 (5)	0.0004 (5)
C13	0.0151 (6)	0.0187 (6)	0.0205 (7)	−0.0008 (5)	0.0019 (5)	0.0004 (5)
C14	0.0183 (7)	0.0326 (8)	0.0282 (8)	−0.0019 (6)	−0.0029 (6)	0.0057 (6)
C15	0.0228 (8)	0.0558 (11)	0.0354 (9)	0.0019 (7)	0.0030 (7)	0.0001 (8)
C16	0.0276 (8)	0.0291 (8)	0.0482 (10)	−0.0131 (6)	−0.0102 (7)	0.0110 (7)
C17	0.0718 (14)	0.0336 (10)	0.0585 (12)	−0.0136 (9)	−0.0296 (10)	−0.0004 (9)
C18	0.0293 (8)	0.0163 (7)	0.0375 (8)	0.0002 (6)	0.0038 (6)	−0.0029 (6)
C19	0.0392 (9)	0.0311 (8)	0.0402 (9)	0.0072 (7)	0.0026 (7)	−0.0003 (7)
C20	0.0267 (7)	0.0185 (7)	0.0357 (8)	−0.0064 (6)	0.0037 (6)	−0.0004 (6)
C21	0.0364 (9)	0.0316 (9)	0.0440 (10)	−0.0022 (7)	0.0142 (7)	0.0034 (7)
C22	0.0150 (6)	0.0153 (6)	0.0202 (7)	−0.0015 (5)	0.0009 (5)	−0.0009 (5)
C23	0.0220 (7)	0.0223 (7)	0.0201 (7)	−0.0021 (5)	0.0005 (5)	0.0007 (5)
C24	0.0282 (8)	0.0307 (8)	0.0202 (7)	−0.0042 (6)	0.0068 (6)	−0.0033 (6)
C25	0.0225 (7)	0.0252 (7)	0.0324 (8)	0.0001 (6)	0.0084 (6)	−0.0069 (6)
C26	0.0172 (6)	0.0189 (7)	0.0323 (8)	0.0001 (5)	0.0026 (6)	−0.0005 (5)
C27	0.0153 (6)	0.0168 (6)	0.0220 (7)	−0.0020 (5)	0.0014 (5)	0.0004 (5)
C28	0.0143 (6)	0.0169 (6)	0.0244 (7)	−0.0020 (5)	−0.0004 (5)	0.0021 (5)
C29	0.0249 (7)	0.0219 (7)	0.0181 (7)	0.0006 (5)	0.0029 (5)	−0.0012 (5)
C30	0.0277 (8)	0.0497 (10)	0.0211 (7)	−0.0089 (7)	0.0060 (6)	−0.0027 (7)
C31	0.0218 (7)	0.0338 (8)	0.0254 (8)	−0.0055 (6)	0.0041 (6)	−0.0063 (6)
C32	0.0187 (7)	0.0281 (7)	0.0243 (7)	−0.0015 (5)	0.0025 (5)	−0.0015 (6)
C33	0.0268 (7)	0.0245 (7)	0.0290 (8)	0.0002 (6)	0.0049 (6)	−0.0054 (6)
C34	0.0242 (7)	0.0219 (7)	0.0304 (8)	0.0005 (6)	0.0031 (6)	0.0023 (6)
C35	0.0159 (6)	0.0293 (7)	0.0230 (7)	0.0008 (5)	0.0013 (5)	−0.0013 (6)
C36	0.0177 (6)	0.0246 (7)	0.0243 (7)	−0.0006 (5)	0.0023 (5)	−0.0034 (5)
C37	0.0201 (7)	0.0235 (7)	0.0269 (8)	−0.0023 (5)	0.0037 (6)	0.0010 (5)
C38	0.0377 (9)	0.0229 (7)	0.0413 (9)	−0.0045 (6)	0.0099 (7)	−0.0035 (6)
O5	0.0232 (5)	0.0368 (6)	0.0229 (5)	0.0051 (4)	−0.0004 (4)	0.0013 (4)
C39	0.0315 (8)	0.0323 (8)	0.0229 (7)	0.0023 (6)	0.0019 (6)	0.0018 (6)
C40	0.0296 (8)	0.0501 (10)	0.0300 (8)	−0.0048 (7)	−0.0039 (7)	0.0052 (7)

### Geometric parameters (Å, º)

**Table:**

O1—C1	1.3802 (15)	C18—C19	1.515 (2)
O1—C13	1.3836 (15)	C18—H18A	0.9900
O2—C28	1.2376 (15)	C18—H18B	0.9900
O3—C35	1.3619 (17)	C19—H19A	0.9800
O3—H3	0.8400	C19—H19B	0.9800
O4—C36	1.3726 (16)	C19—H19C	0.9800
O4—C38	1.4324 (17)	C20—C21	1.527 (2)
N1—C3	1.3756 (17)	C20—H20A	0.9900
N1—C16	1.4556 (18)	C20—H20B	0.9900
N1—C14	1.4583 (18)	C21—H21A	0.9800
N2—C11	1.3805 (17)	C21—H21B	0.9800
N2—C20	1.4636 (18)	C21—H21C	0.9800
N2—C18	1.4653 (18)	C22—C23	1.3871 (18)
N3—C28	1.3593 (16)	C22—C27	1.3895 (18)
N3—C29	1.4537 (16)	C23—C24	1.3949 (19)
N3—C7	1.4989 (16)	C23—H23	0.9500
N4—C31	1.2724 (19)	C24—C25	1.396 (2)
N4—C30	1.4603 (18)	C24—H24	0.9500
C1—C6	1.3877 (18)	C25—C26	1.387 (2)
C1—C2	1.3919 (18)	C25—H25	0.9500
C2—C3	1.4068 (18)	C26—C27	1.3916 (18)
C2—H2	0.9500	C26—H26	0.9500
C3—C4	1.4180 (19)	C27—C28	1.4790 (18)
C4—C5	1.3812 (19)	C29—C30	1.524 (2)
C4—H4A	0.9500	C29—H29A	0.9900
C5—C6	1.4041 (18)	C29—H29B	0.9900
C5—H5	0.9500	C30—H30A	0.9900
C6—C7	1.5193 (17)	C30—H30B	0.9900
C7—C8	1.5159 (18)	C31—C32	1.466 (2)
C7—C22	1.5292 (17)	C31—H31	0.9500
C8—C13	1.3887 (18)	C32—C33	1.392 (2)
C8—C9	1.4077 (18)	C32—C37	1.4052 (19)
C9—C10	1.3786 (18)	C33—C34	1.387 (2)
C9—H9	0.9500	C33—H33	0.9500
C10—C11	1.4176 (19)	C34—C35	1.3851 (19)
C10—H10	0.9500	C34—H34	0.9500
C11—C12	1.4021 (18)	C35—C36	1.4078 (19)
C12—C13	1.3952 (18)	C36—C37	1.3865 (19)
C12—H12	0.9500	C37—H37	0.9500
C14—C15	1.518 (2)	C38—H38A	0.9800
C14—H14A	0.9900	C38—H38B	0.9800
C14—H14B	0.9900	C38—H38C	0.9800
C15—H15A	0.9800	O5—C39	1.4317 (17)
C15—H15B	0.9800	O5—H5A	0.8400
C15—H15C	0.9800	C39—C40	1.503 (2)
C16—C17	1.517 (3)	C39—H39A	0.9900
C16—H16A	0.9900	C39—H39B	0.9900
C16—H16B	0.9900	C40—H40A	0.9800
C17—H17A	0.9800	C40—H40B	0.9800
C17—H17B	0.9800	C40—H40C	0.9800
C17—H17C	0.9800		
			
C1—O1—C13	118.17 (10)	C18—C19—H19C	109.5
C35—O3—H3	109.5	H19A—C19—H19C	109.5
C36—O4—C38	116.56 (11)	H19B—C19—H19C	109.5
C3—N1—C16	122.01 (12)	N2—C20—C21	114.11 (12)
C3—N1—C14	120.95 (12)	N2—C20—H20A	108.7
C16—N1—C14	116.92 (11)	C21—C20—H20A	108.7
C11—N2—C20	120.91 (11)	N2—C20—H20B	108.7
C11—N2—C18	120.85 (11)	C21—C20—H20B	108.7
C20—N2—C18	117.79 (11)	H20A—C20—H20B	107.6
C28—N3—C29	122.51 (11)	C20—C21—H21A	109.5
C28—N3—C7	114.25 (10)	C20—C21—H21B	109.5
C29—N3—C7	123.22 (10)	H21A—C21—H21B	109.5
C31—N4—C30	115.75 (13)	C20—C21—H21C	109.5
O1—C1—C6	123.49 (11)	H21A—C21—H21C	109.5
O1—C1—C2	113.88 (11)	H21B—C21—H21C	109.5
C6—C1—C2	122.62 (12)	C23—C22—C27	120.78 (12)
C1—C2—C3	120.94 (12)	C23—C22—C7	128.55 (11)
C1—C2—H2	119.5	C27—C22—C7	110.66 (11)
C3—C2—H2	119.5	C22—C23—C24	117.82 (13)
N1—C3—C2	120.63 (12)	C22—C23—H23	121.1
N1—C3—C4	122.44 (12)	C24—C23—H23	121.1
C2—C3—C4	116.92 (12)	C23—C24—C25	121.23 (13)
C5—C4—C3	120.51 (12)	C23—C24—H24	119.4
C5—C4—H4A	119.7	C25—C24—H24	119.4
C3—C4—H4A	119.7	C26—C25—C24	120.78 (13)
C4—C5—C6	122.88 (12)	C26—C25—H25	119.6
C4—C5—H5	118.6	C24—C25—H25	119.6
C6—C5—H5	118.6	C25—C26—C27	117.76 (12)
C1—C6—C5	116.09 (12)	C25—C26—H26	121.1
C1—C6—C7	122.24 (11)	C27—C26—H26	121.1
C5—C6—C7	121.63 (11)	C22—C27—C26	121.63 (12)
N3—C7—C8	111.39 (10)	C22—C27—C28	108.63 (11)
N3—C7—C6	110.37 (10)	C26—C27—C28	129.73 (12)
C8—C7—C6	110.12 (10)	O2—C28—N3	124.74 (12)
N3—C7—C22	99.66 (10)	O2—C28—C27	128.57 (12)
C8—C7—C22	112.68 (10)	N3—C28—C27	106.70 (11)
C6—C7—C22	112.25 (10)	N3—C29—C30	113.39 (11)
C13—C8—C9	115.62 (12)	N3—C29—H29A	108.9
C13—C8—C7	122.65 (11)	C30—C29—H29A	108.9
C9—C8—C7	121.73 (11)	N3—C29—H29B	108.9
C10—C9—C8	122.73 (12)	C30—C29—H29B	108.9
C10—C9—H9	118.6	H29A—C29—H29B	107.7
C8—C9—H9	118.6	N4—C30—C29	109.02 (12)
C9—C10—C11	120.95 (12)	N4—C30—H30A	109.9
C9—C10—H10	119.5	C29—C30—H30A	109.9
C11—C10—H10	119.5	N4—C30—H30B	109.9
N2—C11—C12	121.63 (12)	C29—C30—H30B	109.9
N2—C11—C10	121.41 (12)	H30A—C30—H30B	108.3
C12—C11—C10	116.95 (12)	N4—C31—C32	124.72 (14)
C13—C12—C11	120.57 (12)	N4—C31—H31	117.6
C13—C12—H12	119.7	C32—C31—H31	117.6
C11—C12—H12	119.7	C33—C32—C37	119.59 (13)
O1—C13—C8	123.02 (11)	C33—C32—C31	119.17 (13)
O1—C13—C12	113.79 (11)	C37—C32—C31	121.24 (13)
C8—C13—C12	123.18 (12)	C34—C33—C32	120.92 (13)
N1—C14—C15	113.99 (12)	C34—C33—H33	119.5
N1—C14—H14A	108.8	C32—C33—H33	119.5
C15—C14—H14A	108.8	C35—C34—C33	119.69 (13)
N1—C14—H14B	108.8	C35—C34—H34	120.2
C15—C14—H14B	108.8	C33—C34—H34	120.2
H14A—C14—H14B	107.6	O3—C35—C34	118.25 (13)
C14—C15—H15A	109.5	O3—C35—C36	121.73 (12)
C14—C15—H15B	109.5	C34—C35—C36	119.98 (13)
H15A—C15—H15B	109.5	O4—C36—C37	125.29 (13)
C14—C15—H15C	109.5	O4—C36—C35	114.45 (12)
H15A—C15—H15C	109.5	C37—C36—C35	120.26 (12)
H15B—C15—H15C	109.5	C36—C37—C32	119.53 (13)
N1—C16—C17	113.44 (14)	C36—C37—H37	120.2
N1—C16—H16A	108.9	C32—C37—H37	120.2
C17—C16—H16A	108.9	O4—C38—H38A	109.5
N1—C16—H16B	108.9	O4—C38—H38B	109.5
C17—C16—H16B	108.9	H38A—C38—H38B	109.5
H16A—C16—H16B	107.7	O4—C38—H38C	109.5
C16—C17—H17A	109.5	H38A—C38—H38C	109.5
C16—C17—H17B	109.5	H38B—C38—H38C	109.5
H17A—C17—H17B	109.5	C39—O5—H5A	109.5
C16—C17—H17C	109.5	O5—C39—C40	109.53 (12)
H17A—C17—H17C	109.5	O5—C39—H39A	109.8
H17B—C17—H17C	109.5	C40—C39—H39A	109.8
N2—C18—C19	114.87 (12)	O5—C39—H39B	109.8
N2—C18—H18A	108.6	C40—C39—H39B	109.8
C19—C18—H18A	108.6	H39A—C39—H39B	108.2
N2—C18—H18B	108.6	C39—C40—H40A	109.5
C19—C18—H18B	108.6	C39—C40—H40B	109.5
H18A—C18—H18B	107.5	H40A—C40—H40B	109.5
C18—C19—H19A	109.5	C39—C40—H40C	109.5
C18—C19—H19B	109.5	H40A—C40—H40C	109.5
H19A—C19—H19B	109.5	H40B—C40—H40C	109.5
			
C13—O1—C1—C6	−4.83 (18)	C3—N1—C14—C15	89.30 (16)
C13—O1—C1—C2	176.30 (11)	C16—N1—C14—C15	−94.71 (16)
O1—C1—C2—C3	177.85 (11)	C3—N1—C16—C17	88.72 (18)
C6—C1—C2—C3	−1.0 (2)	C14—N1—C16—C17	−87.22 (17)
C16—N1—C3—C2	169.76 (13)	C11—N2—C18—C19	83.16 (16)
C14—N1—C3—C2	−14.5 (2)	C20—N2—C18—C19	−89.23 (16)
C16—N1—C3—C4	−11.1 (2)	C11—N2—C20—C21	−76.12 (16)
C14—N1—C3—C4	164.66 (13)	C18—N2—C20—C21	96.26 (15)
C1—C2—C3—N1	178.44 (12)	N3—C7—C22—C23	177.55 (12)
C1—C2—C3—C4	−0.7 (2)	C8—C7—C22—C23	59.38 (17)
N1—C3—C4—C5	−177.39 (13)	C6—C7—C22—C23	−65.64 (17)
C2—C3—C4—C5	1.8 (2)	N3—C7—C22—C27	−1.82 (13)
C3—C4—C5—C6	−1.1 (2)	C8—C7—C22—C27	−119.99 (12)
O1—C1—C6—C5	−177.09 (12)	C6—C7—C22—C27	114.99 (12)
C2—C1—C6—C5	1.68 (19)	C27—C22—C23—C24	0.62 (19)
O1—C1—C6—C7	0.63 (19)	C7—C22—C23—C24	−178.69 (12)
C2—C1—C6—C7	179.41 (12)	C22—C23—C24—C25	0.0 (2)
C4—C5—C6—C1	−0.6 (2)	C23—C24—C25—C26	−0.7 (2)
C4—C5—C6—C7	−178.35 (12)	C24—C25—C26—C27	0.7 (2)
C28—N3—C7—C8	122.41 (12)	C23—C22—C27—C26	−0.62 (19)
C29—N3—C7—C8	−59.14 (15)	C7—C22—C27—C26	178.81 (11)
C28—N3—C7—C6	−114.94 (12)	C23—C22—C27—C28	−179.42 (11)
C29—N3—C7—C6	63.51 (15)	C7—C22—C27—C28	0.01 (14)
C28—N3—C7—C22	3.28 (13)	C25—C26—C27—C22	−0.06 (19)
C29—N3—C7—C22	−178.27 (11)	C25—C26—C27—C28	178.47 (13)
C1—C6—C7—N3	−119.49 (13)	C29—N3—C28—O2	−1.7 (2)
C5—C6—C7—N3	58.12 (15)	C7—N3—C28—O2	176.80 (12)
C1—C6—C7—C8	3.90 (16)	C29—N3—C28—C27	178.09 (11)
C5—C6—C7—C8	−178.50 (11)	C7—N3—C28—C27	−3.45 (14)
C1—C6—C7—C22	130.32 (13)	C22—C27—C28—O2	−178.20 (13)
C5—C6—C7—C22	−52.08 (16)	C26—C27—C28—O2	3.1 (2)
N3—C7—C8—C13	118.10 (13)	C22—C27—C28—N3	2.06 (14)
C6—C7—C8—C13	−4.69 (16)	C26—C27—C28—N3	−176.62 (13)
C22—C7—C8—C13	−130.87 (12)	C28—N3—C29—C30	92.73 (15)
N3—C7—C8—C9	−62.64 (15)	C7—N3—C29—C30	−85.59 (15)
C6—C7—C8—C9	174.57 (11)	C31—N4—C30—C29	124.69 (14)
C22—C7—C8—C9	48.40 (16)	N3—C29—C30—N4	−166.68 (12)
C13—C8—C9—C10	0.04 (19)	C30—N4—C31—C32	−178.98 (12)
C7—C8—C9—C10	−179.27 (12)	N4—C31—C32—C33	−174.37 (14)
C8—C9—C10—C11	−0.4 (2)	N4—C31—C32—C37	5.3 (2)
C20—N2—C11—C12	169.20 (12)	C37—C32—C33—C34	−0.4 (2)
C18—N2—C11—C12	−3.0 (2)	C31—C32—C33—C34	179.27 (12)
C20—N2—C11—C10	−10.84 (19)	C32—C33—C34—C35	−0.5 (2)
C18—N2—C11—C10	177.01 (12)	C33—C34—C35—O3	179.54 (12)
C9—C10—C11—N2	−179.60 (12)	C33—C34—C35—C36	1.7 (2)
C9—C10—C11—C12	0.36 (19)	C38—O4—C36—C37	10.84 (19)
N2—C11—C12—C13	−179.99 (12)	C38—O4—C36—C35	−169.93 (12)
C10—C11—C12—C13	0.04 (19)	O3—C35—C36—O4	0.98 (18)
C1—O1—C13—C8	3.99 (18)	C34—C35—C36—O4	178.74 (12)
C1—O1—C13—C12	−174.79 (11)	O3—C35—C36—C37	−179.74 (12)
C9—C8—C13—O1	−178.28 (12)	C34—C35—C36—C37	−2.0 (2)
C7—C8—C13—O1	1.02 (19)	O4—C36—C37—C32	−179.74 (12)
C9—C8—C13—C12	0.38 (19)	C35—C36—C37—C32	1.1 (2)
C7—C8—C13—C12	179.69 (12)	C33—C32—C37—C36	0.1 (2)
C11—C12—C13—O1	178.35 (11)	C31—C32—C37—C36	−179.54 (12)
C11—C12—C13—C8	−0.4 (2)		

### Hydrogen-bond geometry (Å, º)

**Table:**

*D*—H···*A*	*D*—H	H···*A*	*D*···*A*	*D*—H···*A*
O3—H3···O5^i^	0.84	1.96	2.7052 (15)	148
O5—H5*A*···O2	0.84	1.93	2.7552 (14)	166

Symmetry code: (i) *x*+1/2, −*y*+3/2, *z*−1/2.
